# Investigation of structural and neurobiochemical differences in brains from high-performance and native hen breeds

**DOI:** 10.1038/s41598-023-27517-3

**Published:** 2023-01-05

**Authors:** Kornel Kasperek, Jadwiga Jaworska-Adamu, Aleksandra Krawczyk, Karol Rycerz, Grzegorz Buszewicz, Dominika Przygodzka, Grzegorz Wójcik, Eliza Blicharska, Kamil Drabik, Anna Czech, Łukasz Wlazło, Mateusz Ossowski, Iwona Rozempolska-Rucińska

**Affiliations:** 1 Institute of Biological Basis of Animal Production, University of Life Sciences in Lublin, Akademicka 13, 20-950 Lublin, Poland; 2 Department of Animal Anatomy and Histology, University of Life Sciences in Lublin, Akademicka 12, 20-950 Lublin, Poland; 3 Department of Forensic Medicine, Medical University of Lublin, 20-090 Lublin, Poland; 4 Department of Inorganic Chemistry, Institute of Chemical Sciences, Faculty of Chemistry, University of Maria Curie-Skłodowska, Lublin, Poland; 5 Department of Analytical Chemistry, Medical University of Lublin, Chodźki 4A, 20-093 Lublin, Poland; 6 Department of Biochemistry and Toxicology, University of Life Sciences in Lublin, Akademicka 13, 20-950 Lublin, Poland; 7 Department of Animal and Environmental Hygiene, University of Life Sciences in Lublin, Akademicka 13, 20-950 Lublin, Poland

**Keywords:** Neuronal physiology, Transporters in the nervous system, Neurophysiology, Animal behaviour, Animal physiology

## Abstract

Selection of livestock has not only led to changes in the level of their performance but also modified their behavior. As a result, within a single species, we have to deal with different behaviors of different breeds. In our study, we assumed that the different behaviors within a species are due to differences in the morphology and physiology of behavior-related systems. Two breeds of hens were used as a model: the highly reactive, fearful and high-performance Leghorn breed and proactive, unselected Green-legged Partridge breed. The higher reactivity and fearfulness of Leghorn hens in comparison to the Green-legged Partridge breed may be related to the greater number of neurons in the paraventricular nucleus and anterior hypothalamus and the higher content of zinc and iron in the brain, as these elements are involved in neuronal conduction and myelination processes. The reactive behaviours of Green-legged Partridge hens may be associated with the lower number of neurons in the paraventricular nucleus and the anterior hypothalamus and the higher concentration of dopamine and copper ions in the brain. The analyses confirmed the hypothesis of the existence of interbreed differences in the morphology and physiology of behaviour-related systems, which most probably emerged through unintentional and correlated selection towards high production performance. Consequently, attention should be drawn that the selection of a given genotype (breed) towards a specific environment could lead to creation of highly specialised lines that may not achieve homeostasis in every maintenance system.

## Introduction

In the process of domestication and breeding of hens (*Gallus* *gallus* *domesticus*), many breeds characterised by different performance values and different behaviour patterns were produced. As reported by Agnvall and Jensen^1^ in their study on the selection of the red junglefowl towards reduced fearfulness, species-specific behaviour is generally resistant to changes occurring during domestication. However, since personality in poultry exhibits a certain degree of heritability^2–4^, it should be assumed that the multiyear selection pressure may have led to a correlated and unintended modification of bird behaviour. As suggested by Mehlhorn and Petow^5^, breeding and strong selection towards high egg production is accompanied by unintentional selection towards smaller brains. Other authors have proved that even differences in maintenance systems may have a limited but noticeable effect on the development of the CNS^6^ (Central Nervous System). The selection of a given genotype towards a specific environment has resulted in creation of highly specialised lines that may not achieve homeostasis in every maintenance system due to genotype-environment interactions^7–9^. The most frequent consequences of the disparity between the genotype and the maintenance system are behavioural disorders leading to reduced productivity and welfare^10^. Hence, it is important to assess the breeding material correctly in terms of behaviour in order to match a given genotype with the optimal production system. Therefore, poultry rearing norms that are standardized for the species will not guarantee welfare in every case (production system).

Previous studies demonstrated differences between the behaviour of the low-performance native chicken breed, i.e. the Green-legged Partridge, and the intensively selected high-performance Leghorn breed^11–14^. Compared to the Green-legged Partridge breed, the Leghorn line was characterised by greater physical activity and exploration after reaching sexual maturity^13,14^, which was not observed in the chicks up to the age of 21 days^12^. The high activity of Leghorn hens and their considerable exploration need were reflected in their short-term interest in the explored objects^13,14^. Moreover, in comparison with the Green-legged Partridge breed, Leghorn hens can be characterized as fearful and restless^11–14^. These results prompted the need to analyse the possible causes of such evident behavioural differences between the breeds.

We assumed that such visible interbreed behavioural differences between Leghorn and Green-legged Partridge hens have their origins in the anatomy and physiology of systems involved in the behaviour of these birds. Therefore, the aim of the study was to compare certain behaviour-related anatomical and physiological traits in two hen breeds differing in their behaviour patterns.

## Results

### Morphological and morphometric analyses

**Figure 1:**
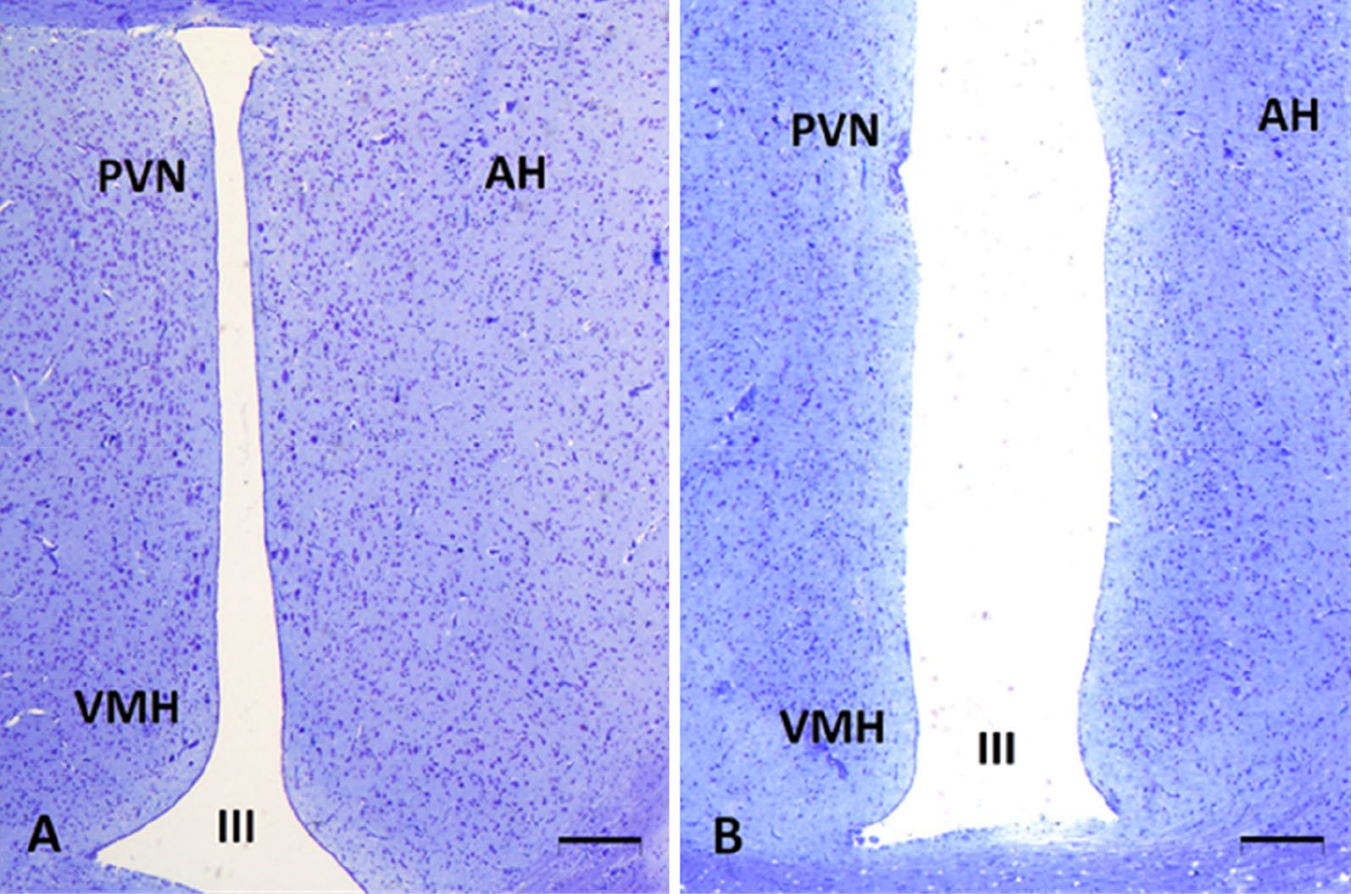
Ventromedial hypothalamus (VMH), paraventricular nucleus (PVN), and anterior hypothalamus (AH) of Leghorn (**A**) and Green-legged (**B**) hens; *III* third ventricle of the brain (cresyl violet staining, scale bar 200 µm).

**Figure 2:**
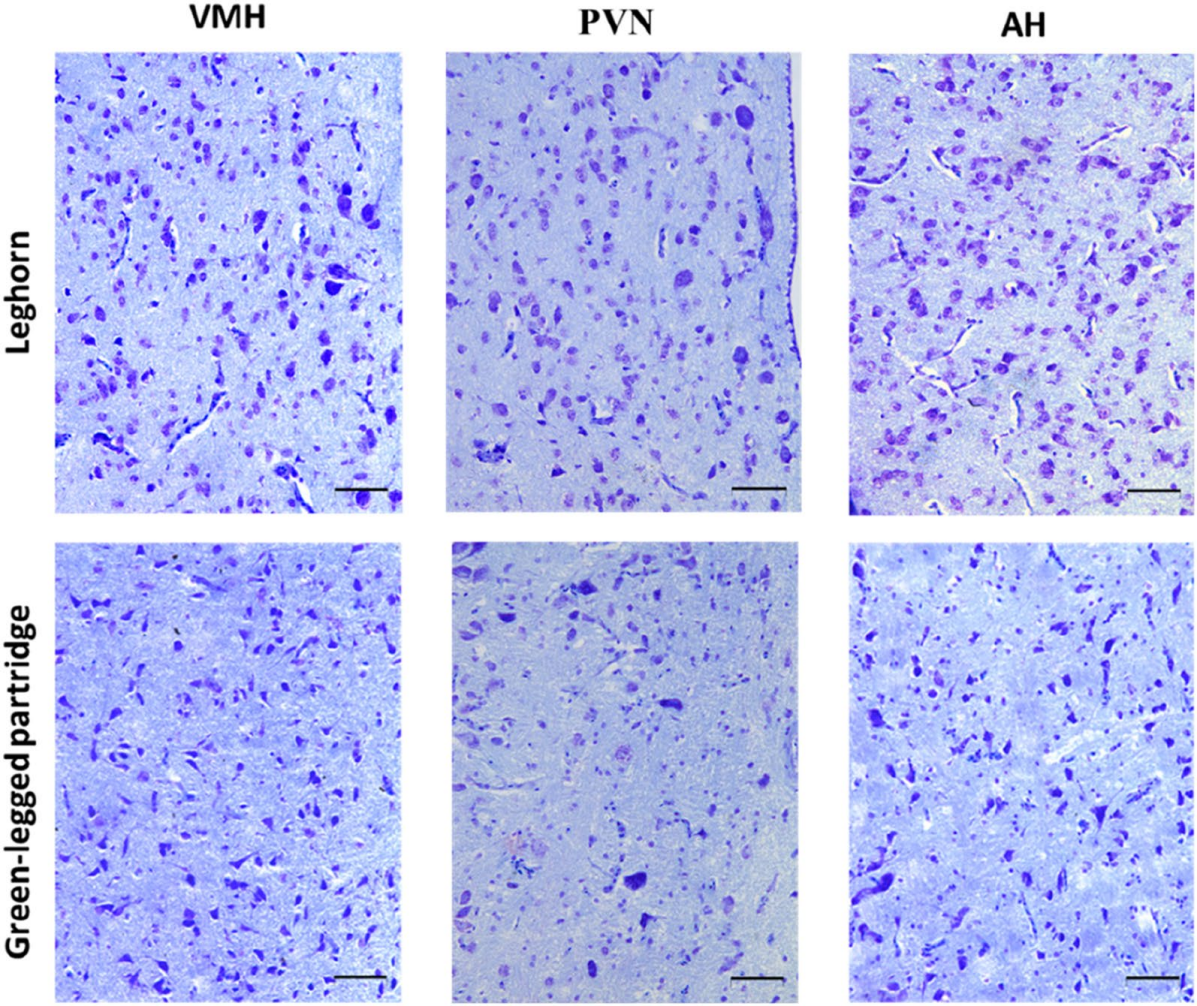
Ventromedial hypothalamus (VMH), paraventricular nucleus (PVN), and anterior hypothalamus (AH) of Leghorn and Green-legged Partridge hens (cresyl violet staining, scale bar 50 µm).

**Figure 3:**
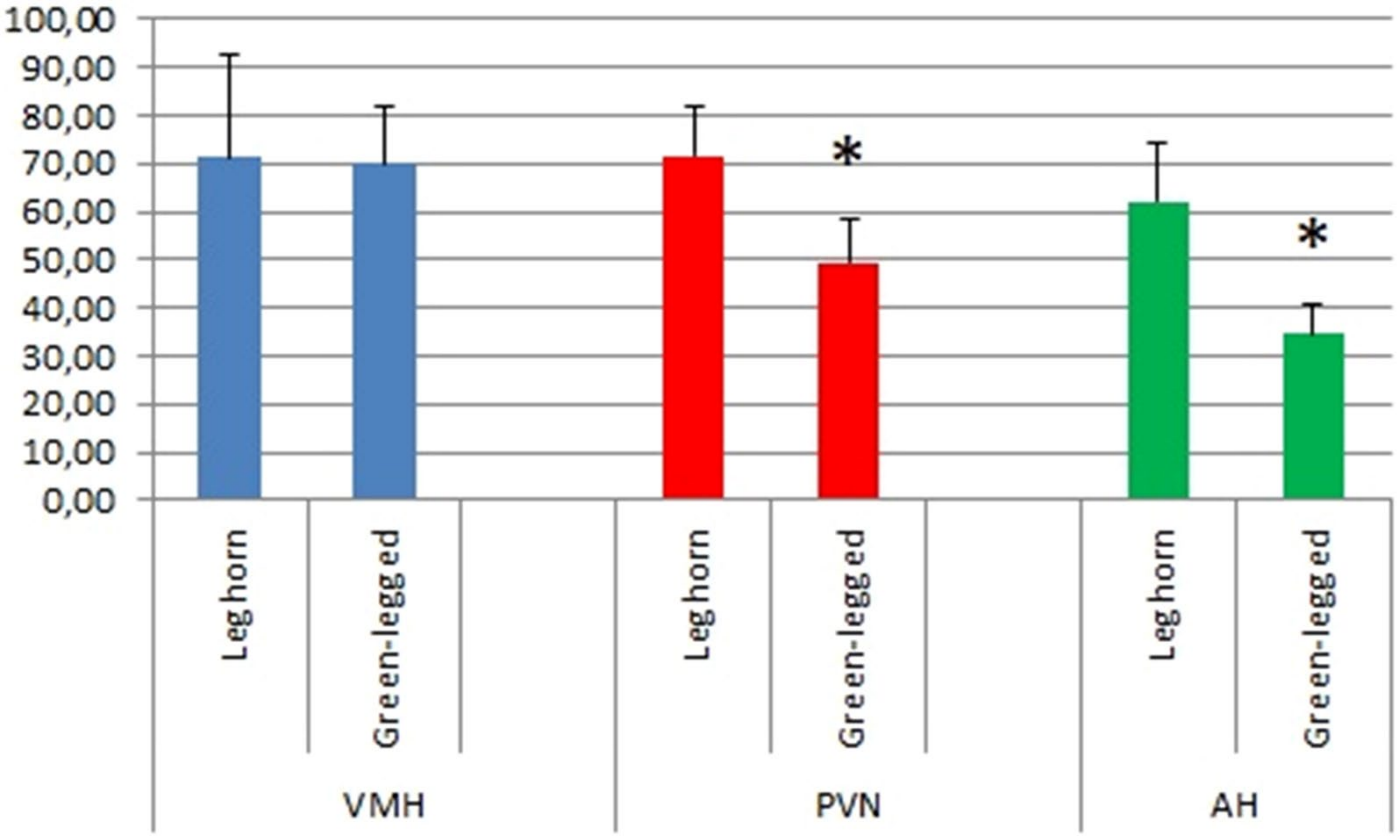
Number of neurons in the VMH, PVN, and AH of Leghorn and Green-legged hens. Data show the mean number of neurons with standard deviation in an area of 6.25 × 10^–2^ mm^2^. *Statistically significant differences at p < 0.05 (ANOVA).

In the Leghorn and Green-legged Partridge hens, we demonstrated the presence of multiform nervous cells of different sizes in the studied areas (Fig. 1). We demonstrated multipolar neurons of similar sizes but with different shapes (oval, round, triangular, and fusiform) in the ventromedial hypothalamus (VMH), the paraventricular nucleus (PVN), and the anterior hypothalamus (AH) in both breeds of hens. We observed large, oval, and triangular cells in VMH of Leghorn hens. Similar neurons were present in PVN, especially near the third ventricle of the brain in both breeds of hens (Fig. 2). The morphometric analyses did not show statistically significant differences in the number of neurons in VMH between the two studied breeds of hens. However, the number of nerve cells in PVN and AH was statistically greater in the Leghorn hens (Fig. 3).

### Microelements in the brain

No significant differences in the content of most of the analysed microelements were found between the Leghorn and Green-legged Partridge breeds (Table 1). However, it should be noted that high standard deviations of the means were found in the case of cobalt, nickel, strontium, cadmium, barium, lead, and mercury, which undermines the reliability of inference of the differences in the content of these elements between the breeds. The brain tissue from the Leghorn birds exhibited a significantly higher concentration of iron, zinc, and molybdenum than the brains of the Green-legged Partridge chickens (P = 0.002, P < 0.001, and P = 0.007, respectively). In turn, the concentration of copper was significantly higher in the brain of the Green-legged Partridge birds than in the Leghorn brain tissue (P = 0.002) (Table 1).

**Table 1:** Content of microelements in the brain of Leghorn (H-33) and Green-legged Partridge (Zk) hens—mean (mg/100 g) ± standard deviation (SD).

Microelements	Leghorn	Green-legged Partridge	Pr. > F
	Mean ± SD	Mean ± SD	
Aluminium	0.071 ± 0.046	0.067 ± 0.046	0.83
Vanadium	0.0048 ± 0.0009	0.0045 ± 0.0005	0.37
Chromium	0.055 ± 0.012	0.051 ± 0.005	0.17
Manganese	0.191 ± 0.013	0.198 ± 0.013	0.49
Iron	7.27 ± 0.67	6.47 ± 0.52	0.002
Cobalt	0.004 ± 0.003	0.005 ± 0.009	0.73
Nickel	0.016 ± 0.014	0.025 ± 0.019	0.23
Copper	0.92 ± 0.11	1.2 ± 0.23	0.002
Zinc	4.42 ± 0.3	3.99 ± 0.21	< 0.001
Arsenic	0.0018 ± 0.0004	0.0019 ± 0.0004	0.72
Selenium	0.081 ± 0.012	0.077 ± 0.006	0.38
Strontium	0.061 ± 0.05	0.078 ± 0.14	0.74
Cadmium	0.0003 ± 0.0002	0.0006 ± 0.0004	0.27
Barium	0.033 ± 0.017	0.03 ± 0.019	0.84
Palladium	0.0043 ± 0.003	0.0031 ± 0.002	0.31
Molybdenum	0.037 ± 0.004	0.033 ± 0.003	0.007
Mercury	0.024 ± 0.021	0.019 ± 0.008	0.55

### Neurohormones

In the case of biogenic amines, the concentration of dopamine was significantly higher in the brain of the Green-legged Partridge birds than in the Leghorn hens (Table 2). The content of this neurotransmitter in the plasma did not differ between the breeds. There were no differences in the serotonin content between the breeds, regardless of the type of tissue. The content of glucocorticosteroids (corticosterone, cortisol, and cortisone) in both plasma and brain tissue was similar in both breeds. These compounds exhibited only a tendency towards a greater amount in the blood plasma of H-33 vs. Zk and a higher concentration in the brain of Zk vs. H-33 (Table 2).

**Table 2:** Mean ranks of the content of neurotransmitters in the plasma and brain of Leghorn (H-33) and Green-legged Partridge (Zk) hens (Mann–Whitney *U* test).

Hormones	Plasma			Brain		
	Mean ranks in Mann–Whitney *U* test		Pr. > = |S|	Mean ranks in Mann–Whitney *U* test		Pr. > = |S|
	H-33	Zk		H-33	Zk	
Dopamine	9.16	7.22	0.46	4.2	8.75	0.045
Serotonine	8.14	9.6	0.61	9.43	7.78	0.54
Corticosterone	7.5	5.5	0.39	4.37	7.56	0.16
Cortisol	5	2	0.1	5	6.37	0.63
Cortisone	5.1	1.9	0.12	4.16	6.69	0.29

### White blood cells

There were no differences in the white blood cell profile between the Leghorn and the Green-legged Partridge hens (Table 3).

**Table 3:** Analysis of the significance of differences in the number of white blood cells in Leghorn (H-33) and Green-legged Partridge (Zk) hens—mean ± standard deviation (SD).

Breed	Leghorn	Green-legged Partridge	Pr. > F
Trait	Mean ± SD	Mean ± SD	
Leukocytes (10^9^/l)	19.1 ± 3.2	15.6 ± 4.9	0.17
Heterophils (%)	43 ± 10.1	46.5 ± 13.7	0.62
Lymphocytes (%)	48.7 ± 12.5	45.2 ± 11.2	0.62
Monocytes (%)	8 ± 3.2	8 ± 2.9	1
H:L ratio	0.98 ± 0.49	1.16 ± 0.63	0.61

### Intestinal microbiota

The microbiological analyses of the intestinal contents revealed a significantly higher number of colonies of anaerobic *Clostridium* *perfringens* in the large intestine and caecum of the Leghorn hens (Table 4). Greater abundance of coliform bacteria and *Escherichia* *coli* was detected the large intestine and caecum of the Green-legged Partridge birds. The content of fungi was higher only in the small intestine in the Green-legged Partridge hens. The total number of lactic acid *Lactobacillus* bacteria was higher in the alimentary tracts of the Leghorn hens but did not differ in the individual segments of the alimentary tract. A greater number of mesophilic bacteria and *Listeria* species was detected the caecum of the Green-legged Partridge hens (Table 4). The analysis showed no presence of bacteria of the genus *Salmonella*.

**Table 4:** Content of selected microorganisms in the intestinal microflora in Green-legged Partridge (Zk) and Leghorn (H-33) hens—means ± standard deviation (SD).

	Intestine						Effect of: (*P* value)		
	Small		Large		Caecum				
	Zk	H-33	Zk	H-33	Zk	H-33	B	I	B × I
Total number of mesophilic aerobic bacteria	5.97^ab^ ± 0.31	5.5^bc^ ± 0.4	6.48^a^ ± 0.21	6.34^a^ ± 0.25	6.41^a^ ± 0.29	5.28^c^ ± 0.34	< 0.001	< 0.001	0.01
Total number of fungi	3.58^a^ ± 0.38	2.68^b^ ± 0.02	3.18^ab^ ± 0.11	3.53^ab^ ± 0.49	4.07^a^ ± 0.46	3.93^a^ ± 0.57	0.17	< 0.001	0.017
Total number of coliform bacteria	2.23^d^ ± 0.22	2.04^d^ ± 0.15	6.15^a^ ± 0.24	4.26^c^ ± 0.23	6.24^a^ ± 0.24	5.54^b^ ± 0.18	< 0.001	< 0.001	< 0.001
*E.* *coli*	2.55^d^ ± 0.13	1.49^e^ ± 0.13	6.29^a^ ± 0.09	4.25^c^ ± 0.08	6.66^a^ ± 0.67	5.08^b^ ± 0.18	< 0.001	< 0.001	< 0.001
Total number of *Clostridium* *perfringens*	2.97^c^ ± 0.02	2.8^c^ ± 0.02	5.67^b^ ± 0.12	6.44^a^ ± 0.03	5.9^b^ ± 0.24	6.63^a^ ± 0.01	< 0.001	< 0.001	< 0.001
Total number of lactic acid bacteria of the genus *Lactobacillus*	7.03^bc^ ± 0.59	7.12^bc^ ± 0.71	6.61^c^ ± 0.12	7.28^bc^ ± 0.54	7.98^ab^ ± 0.68	8.96^a^ ± 0.03	< 0.001	< 0.001	0.23
*Listeria* spp.	3.79^ab^ ± 0.45	2.94^b^ ± 0.02	3.78^ab^ ± 0.55	3.03^b^ ± 0.12	4.4^a^ ± 0.51	3.32^b^ ± 0.48	< 0.001	0.048	0.73

^a,b^Means in the rows differ significantly at p ≤ 0.05 (Tukey test). *B* breed, *I* section of the intestine.

## Discussion

Neurons are the basic processing units of the brain; hence, it was concluded through simple inference that the larger the brain, the greater the number of neurons and the better the cognitive abilities^15–18^. Consequently, in comparative and cognitive neuroscience, the size of the brain is the dominant measure of its functional capacity. However, recent studies have shown that the brain mass may be less tightly correlated with the number of neurons than previously thought, as vertebrate brains of similar size have been shown to differ in the number, density, and distribution of neurons in different brain regions^19,20^. As reported by Olkowicz et al.^20^, the brain of a parrot or a songbird has on average twice as many neurons as the same-sized brain of a primate; hence, the brain of a bird may provide considerably higher “cognitive power” per unit mass than a mammalian brain. The same authors^20^ proved that the emu, the red junglefowl, and the domestic pigeon generally have fewer neurons than songbirds and parrots. The authors suggest that the greater number of neurons in the parrot and songbird brains than in e.g. the red junglefowl is related to the fact that post-hatching neurogenesis and brain maturation are associated with the emergence of specialised circuits mediating voice learning, which is crucial in birds with an extensive vocal repertoire^20^. The present study has proved that the density of neurons may differ even within one species and may result from interbreed differences. The present analysis of three brain regions showed no differences between the breeds in the number of neurons in the ventromedial hypothalamus (VMH). Additionally, a significantly higher density of neurons was detected in the paraventricular nucleus (PVN) and the anterior hypothalamus (AH) in the brain of the Leghorn vs. Green-legged Partridge hens. Appetite is modulated by various regions of the CNS, and the most important role is assigned to the hypothalamus^21,22^. The ventromedial hypothalamus (VMH) and the paraventricular nucleus (PVN) analysed in the present study are examples of hypothalamic subregions involved in the expression of the feeding behaviour^23^. As suggested by Buntin et al.^24^, the ventromedial hypothalamus (VMH) in birds is the most important brain centre responsible for the regulation of food intake. This function is related to the activity of prolactin, however, the authors emphasise that prolactin is active in other brain regions as well, and VMH integrity is not essential for the expression of hyperphagia^24^. Although we found no differences in the number of neurons in VMH between the H-33 and Zk breeds, the H-33 hens had a significantly greater number of neurons in the paraventricular nucleus (PVN) than the Zk birds. As reported by Yousefvand and Hamidi^23^, the information about satiety or lack of satiety reaches the paraventricular nucleus first and is then transmitted by various neurotransmitters and neuromodulators. Hence, the interbreed differences found in the present study suggest that the morphological traits of Leghorns may indicate greater activity of their brain regions responsible for food intake, compared to the Green-legged Partridge breed. This is a sensible suggestion, given the much higher food demand exhibited by the Leghorn breed, which is highly productive and lays almost twice as many eggs as Green-legged Partridge hens. Assuming a feed conversion rate exceeding 120 g of feed/egg^25^ and the higher (by 140 eggs) productivity of Leghorn hens than that of the Zk breed, the feed intake for egg production in the first year of life by the former breed is by approx. 17 kg higher. It can also be assumed that the higher motor activity of H-33 compared to Zk reported in previous studies^11–14^ may increase the demand for energy and, consequently, feed. Another important role of the paraventricular nucleus (PVN) is the activation of the HPA (hypothalamic–pituitary–adrenal) axis, as the corticotropin releasing hormone (CRH)-containing neurons in the PVN support the early response of the HPA axis to stress^26^. CRH is synthesised in paraventricular nucleus (PVN) neurons in the hypothalamus and released in the external zone of the median eminence. CRH is well known to be involved in the autonomous^27^ and behavioural stress response^28,29^. The greater number of neurons in PVN may explain the greater fearfulness of the Leghorn breed reported in previous studies^13,14^, in comparison to the Green-legged Partridge line. Studies conducted on songbirds have revealed that the number of dorsal AH neurons is positively correlated with aggression^30^. The greater number of AH neurons in the Leghorn brain may indicate stronger aggressive behaviour than that of the Green-legged Partridge hens. In our previous behavioural studies, we did not analyse the Zk and H-33 breeds in terms of aggressive behaviour, but we showed high motor activity of Leghorns, which may be indirectly related to the higher frequency of aversive behaviours in this population. Additionally, in comparison with brown hens, white laying hens have been found to be reactive and exhibit a high hormonal and behavioural response to stress^31–34^. Other studies have evidenced higher aggressiveness in Leghorns than in red junglefowl, which was explained by the poorer social learning ability and the weaker ability to cope with disturbances in the group in comparison with the ancestral breed^35^. The Green-legged Partridge line analysed in this study is regarded as a primitive breed; hence our results correspond with the findings reported by Väisänen et al.^35^. However, the association of the greater number of neurons in AH with the greater intensity of aggressive behaviour requires further research, as Dennis and Cheng^36^ demonstrated differences in the neurotransmitter regulation of aggression between high and low aggressive chicken lines due to the involvement of different receptor systems.

The homeostasis of biometallic microelements, e.g. such as copper, iron, manganese, and zinc, is crucial for the structure and functioning of the CNS^37^. Although the birds analysed in the present study were maintained in the same environmental and nutritional conditions throughout life, there were differences in the content of elements in their brains. The brain of the Green-legged Partridge hens contained significantly higher levels of copper than the brain tissue of the Leghorn birds. Copper is essential for proper functioning of the nervous system, and changes in brain copper homeostasis cause neurological disorders. In the brain, copper is essential for synaptogenesis processes. Copper is essential for brain-specific enzymes that control such neurotransmitters as dopamine as well as food neuropeptides and amines^38^. Due to the complex functions of copper in the CNS, the interbreed differences in the content of this element in poultry require further detailed analyses of different regions of the brain. Other differences noted between the hen breeds were the significantly higher concentrations of zinc, iron, and molybdenum. The largest amounts of zinc in the organism are deposited in the brain, where the element ensures proper nerve conduction, and zinc ions are located near the presynaptic neuron endings. Since zinc influences neurotransmission and sensory processing and activates neuronal signalling pathways^39^ and the higher density of neurons was observed in the PVN and AH of the brain in the Leghorns, the higher content of this element in the samples seems to be confirmed. As reported by Golub et al.^40^, many studies have demonstrated that zinc deficiency in the diet of animals leads to behavioural disorders, which are mainly manifested by reduced motor and cognitive activity. These reports^40^ suggest association of the higher activity and fearfulness of Leghorn hens with the higher number of neurons and the higher concentration of zinc ions. The highest amounts of iron are accumulated in brain and liver tissues. In the brain, iron is the second most abundant metal after zinc and is essential for the myelination process and in the metabolism and synthesis of neurotransmitters^41^. The higher content of this element in the Leghorn hens should also be associated with the brain of these birds, which has a greater number of neurons than the brain of Green-legged Partridge hens. Molybdenum is an essential element supporting brain functions, and disturbances in its homeostasis result in molybdenum cofactor type A deficiency, which manifests itself in the early postnatal period with severe seizures in humans^42^. The role of molybdenum in the nervous system function has not been fully explored; hence, the higher content of this element in the brains of Leghorn vs. Green-legged Partridge hens has not been elucidated yet.

Animal welfare is increasingly being defined as the absence of negative experiences and primarily by the presence of positive experiences e.g. pleasure^43,44^. Despite this approach, most physiological measurements assessing animal welfare are based on stress-related parameters, e.g. the level of glucocorticosteroids, assuming that the absence of a negative effect is an indicator of well-being^44^. However, there are markers that can serve as positive indicators in assessment of animal welfare, i.e. serotonin and dopamine^43,45,46^. Based on the results of the present analyses of both hen breeds, i.e. the higher content of dopamine in the brain, it can be concluded that the Green-legged Partridge hens exhibited a higher level of welfare. Despite the differences in the content of dopamine in the brain, no such discrepancies were noted in the analysis of the blood plasma. This was probably related to the inability of dopamine to cross the blood–brain barrier; hence, dopamine synthesis and functions in peripheral areas are largely independent of its synthesis and activity in the brain^47^. Dopamine improves the ability to cope with fear and stress and plays a key role in the neurophysiological association with pterophagy^48^. A number of studies have indicated lower plasma dopamine levels in birds characterised by increased pterophagy^49–51^. Van Hierden et al.^48^ found a lower dopamine and serotonin turnover in the brain of chicks from a line selected towards high feather pecking (HFP), in comparison with a low feather pecking (LFP) line, which is consistent with the results of studies on adult pigs^52^ and rodents^53^. This also confirms the assumption that HFP and LFP lines that represent proactive and reactive coping strategies, respectively. Given the present results and those reported by Van Hierden et al.^48^, it can be concluded that Leghorn hens represent proactive birds with an increased risk of pterophagy. This is confirmed by our behavioural studies, where Leghorns were defined as more active and fearful than Green-legged Partridge chickens^13,14^. Possibly, the lower concentration of dopamine in Leghorn hens is a result of a correlated and unintentional selection because, as reported by Ahmed-Farid et al.^54^, laying hens selected for increased productive performance and survival exhibit a lower concentration of dopamine in the blood than hens selected towards low productivity and survival. At this point, it is also worth noting that the higher dopamine content in Green-legged hens may be directly associated with the higher content of copper in the brain, which is directly involved in the synthesis of this neurotransmitter^38^. Furthermore, as reported by Van Hierden et al.^48^, the dopamine and serotonin turnover decreases with stress, whereas the content of corticosteroids increases. In the present study, the birds differed only in the content of dopamine in the brain, but there were no interbreed differences in the content of the other neurotransmitters in the brain and plasma. Noteworthy, no stress factor was included in the present study, and the birds were assessed only on the basis of interbreed differences. Moreover, as suggested by Dennis and Cheng^36^, birds with high and low aggressiveness levels may differ in the regulation of neurotransmitters by different receptor systems, which largely complicates the interpretation of neurotransmitter-receptor interactions in different breeds. Thus, the absence of differences between the levels of serotonin and corticosteroids (corticosterone, cortisol, and cortisone) does not necessarily indicate the same emotional state of the analysed breeds. Here, the study conducted by Ericsson and Jensen^55^ can be cited, as the authors concluded that the HPA system (hypothalamic–pituitary–adrenal axis) is less sensitive in domestic chickens than in red junglefowl, despite the higher corticosterone response.

The white blood cell count indicated no differences between the H-33 and Zk lines. Nevertheless, an earlier study^11^ carried out on the same chicken lines indicated that Zk birds were less susceptible to stress than H-33 chickens due to a lower H:L ratio and a higher level of lysozyme. However, the interpretation of the results obtained for the leukocyte count and the H:L ratio may pose some difficulties^56,57^. A high level of heterophils in blood and thus a high H:L ratio may indicate an ongoing disease in the organism or, on the contrary, may prove a very high efficiency of the immune system, which is reflected in a quick response to the presence of the pathogen. In turn, a high number of lymphocytes and thus a lower H: L ratio may indicate the absence of a disease in the system or the response of the organism to a stressful situation. Moreover, the birds studied by Rozempolska-Rucińska et al.^11^ were at a peak of their lying performance (33 weeks of age), whereas the birds included in the present study had a completely different hormonal status, as they were analysed after the production period at 58 weeks of age. It is well known that age and feathering have an impact on the H:L value as well^58,59^. The absence of differences in the H:L ratio between the breeds corresponds to the absence of differences in the content of corticosteroids in the brain and blood of both breeds. It has been shown that there is a high positive correlation between the H:L ratio and the content of corticosterone in blood^60^. Consequently, the level of glucorticosteroids and the white blood cell profile shown in the present study do not indicate differences in the behaviour of the analysed H-33 and Zk lines. Nevertheless, this result should be treated with caution, as the glucorticosteroid level and the H:L ratio change under the influence of stressors, which were not induced artificially in this study. It is probable that interbreed differences might be visible in stressful conditions.

The communication between the brain and the intestinal microbiota has been defined as the microbiota–gut–brain (MGB) axis, and studies of this relationship indicate that its dysregulation induces fear-related and stress behaviours^61,62^. Stressful and emotional situations have an impact on the intestine physiology and the living environment of microbiota through the release of stress hormones or sympathetic neurotransmitters^63^. In the composition of the intestinal microflora presented in this study, bacteria from the *Lactobacillus* group can be regarded as microorganisms exerting a potentially positive effect on the CNS, which has been evidenced in animal models^64–66^. It has been reported that chickens with pterophagy behavioural disturbances had a greater amount of *Clostridiales* and lower amounts of *Staphylococcus* and *Lactobacillus* than laying hens that did not show pterophagy behaviours^67,68^. In both hen breeds, similar numbers of *Lactobacillus* bacteria were detected in the individual segments of the alimentary tract, but the total number of the microorganisms was generally higher in the Leghorn line. In terms of the MGB axis, *Clostridium* *perfringens*, *Escherichia* *coli*, and *Listeria* spp. can be regarded as microorganisms exerting adverse effects on the CNS. In the analysed alimentary tract segments, *Clostridium* *perfringens* was more abundant in the Leghorn line, whereas higher *Escherichia* *coli* and *Listeria* spp. numbers were detected in the intestines of the Green-legged Partridge hens. Taking into account all the analysed intestinal microorganisms, it can be concluded that the Leghorn line exhibits a better microflora composition for the function of the MGB axis despite the greater abundance of *Clostridium* *perfringens*. Given the generally higher content of lactic acid bacteria in the Leghorn group, it may be assumed that these birds have higher levels of serotonin, i.e. one of the neuroactive substances produced by the microbiota exerting a “positive” effect on the CNS after crossing the blood–brain barrier^65,69^. However, no significant differences in the content of serotonin were found in the blood and brain tissues of the H-33 and Zk lines. Probably, the absence of such a relationship was associated with the fact that the differences in the microbiome between H-33 and Zk are small and difficult to elucidate, given the potential interactions between the components of the intestinal flora. Moreover, as reported by Mayer et al.^70^, the relationship between the intestinal microflora and the CNS is influenced by many factors, e.g. time, breed/genetic strain, sex, and species, which have not yet been fully explored. It should also be highlighted that most investigations of the impact of the microbiome on the CNS consisted in experimental induction of stress and analyses of changes in the microbiome, unlike in the present study. Studies of H-33 and Zk populations with experimental inclusion of a stress factor may provide clearer results, as stress and emotions exert an effect on the intestinal physiology and the living environment of the microbiota^63,71,72^.

The proactive behaviours of Leghorn (H-33) hens in comparison with the reactive behaviours of Green-legged Partridge (Zk) individuals defined in behavioural studies were confirmed by the selected traits of the organism that may potentially influence hen’s behaviour. The higher activity and fearfulness of H-33 in comparison to Zk may be related to the higher number of neurons in PVN and AH and the higher content of zinc and iron in the brain, i.e. elements involved in neuronal conduction and myelination processes. The reactive behaviour of the Green-legged Partridge hens may be associated with the lower number of neurons in PVN and AH and the higher concentration of dopamine and copper ions in their brain. A rather unclear picture of the previously confirmed behaviour patterns in the hen breeds was provided by the analysis of the microbiome, the serotonin and glucocorticoid content, and the H:L ratio. However, these markers change noticeably in response to stress, which deliberately was not employed in this study. In conclusion, the study results support the hypothesis of the interbreed differences in the morphology and physiology of behaviour-related systems, which most likely result from the process of unintentional and correlated selection towards high production performance of hens. As a consequence of the results obtained, generalizing welfare conditions at the species level seems to be too much of a generalization since significant differences in behavior and its neurobiological determinants already exist at the level of breeds and lines.

## Materials and methods

The material for this study was taken from livestock, after their scheduled slaughter, following the end of laying production. According to "Directive 2010/63/EU of the European Parliament and of the Council of September 22, 2010 on the protection of animals used for scientific purposes," it is not a procedure to kill an animal for the sole purpose of using its organs or tissues for the purposes specified in the directive, so approval was not sought from the ethics committee. The number of birds from which tissues were collected for scientific purposes was reported to the Local Ethical Committee, Lublin, Poland.

In total, 45 Leghorn hens (H-33) and 45 Green-legged Partridge hens (Zk) were used in the study. The H-33 Leghorn line is selected as a component for the production of high-performance layer hybrids. The Leghorns analysed in the study are kept in intensive egg production systems, with an annual laying performance of approx. 300 eggs. The Green-legged Partridge is a native Polish breed used mainly in extensive systems. Its laying performance is approx. 160 eggs in the first production year. The Zk line is the oldest closed population of this breed with its flocks included in the genetic resource conservation program. Hence, Zk hens are not selected towards increased egg production, and the population is reared in accordance with the breed pattern and with origin control. Only female birds were included in the study. During the rearing and production periods, the birds of both breeds were kept in the litter system, in the same conditions, with the same feeding program, and at the same density. The birds were reared in the same building, in two separate boxes with an artificial lighting regime for 18 weeks. After 18 weeks, the birds of both lines were moved to separate boxes of the production building, where they were kept until 58 weeks of age. The material for analyses was collected post-mortem from the birds after the end of the production (58 weeks of age). Birds with no visible loss of feathers and with a body weight similar to the average weight in the population: 1750 ± 50 g (Green-legged Partridge) and 1900 ± 50 g (Leghorn) were selected for slaughter. In total, 45 brains were dissected as individual samples from each breed: 5 brains for morphological and morphometric analyses, 10 brains for determination of the composition of microelements, and 10 brains for determination of the content of dopamine, serotonin, corticosterone, cortisol, and cortisone. Brains intended for the morphological and morphometric analyses were preserved in 5% formalin, whereas brains for the determination of the content of microelements and neurohormones were frozen in liquid nitrogen. Brain samples for the trace element analysis were collected in accordance with the analytical protocol. A Suprapure nitric acid solution (5% (v/v)) was used to disinfect all materials that were in contact with the tissue samples. The brain was removed from the skull, washed with ultrapure water (Milli-Q, Millipore, Raleigh, NC, USA; resistance: 18.2 MΩ-cm) to reduce the probability of blood contamination of the sample, and freeze-dried. Blood was collected during slaughter from 40 individuals (20 per breed): 10 samples were intended for morphological analysis and 10 samples were used for the determination of the levels of dopamine, serotonin, corticosterone, cortisol, and cortisone. Blood for morphological analyses was collected in 4-ml test tubes containing disodium salt of ethylene diaminetetra-acetic acid (EDTA), and blood samples intended for biochemical assays were kept in 4-ml tubes with lithium heparin. Alimentary tracts were dissected from 5 birds per breed for microbiological analyses. The brains, blood samples, and alimentary tracts were collected from the same birds.

### Morphological and morphometric analyses

Leghorn and Green-legged Partridge hen brains were collected, embedded in paraffin blocks, and cut into 10-µm thick frontal sections in a microtome. The slides were stained with cresyl violet. The ventromedial hypothalamus (VMH), paraventricular nucleus (PVN), and anterior hypothalamus (AH) were analysed and photographed under the Olympus BX51 light microscope (Olympus, Tokyo, Japan) with a digital camera (Olympus Color View III). Morphometric assessment was conducted on randomly selected 20 microphotographs from every animal and every studied area. Neurons were counted in a grid of 6.25 × 10^–2^ mm^2^. Data were presented as means with standard deviation.

### Microelements in brain tissue

The content of elements was determined in lyophilisates consisting of the brain and the cerebellum. To this end, each sample was weighed and transferred into sealed EasyPrep Teflon vessels, flooded with 5 cm^3^ of concentrated nitric acid (V) 65% (Suprapur, Merck) and 1 cm^3^ of 30% hydrogen peroxide (ROMIL SpA Super Purity). Next, the sample was digested at 180 °C in a Mars 6 microwave digestion system (CEM, Matthews, Matthews). After this process, the mineralisate was diluted with deionised water in flasks to a total volume of 350 cm^3^. All samples were analysed using an Agilent 7700 Series ICP-MS spectrometer. The water used for the determinations was provided by the Hydrolab water demineralisation system HLP10. Standards for the calibration curves were prepared from ROMIL solutions (HT20). The microelements were analysed using the following isotopic masses: ^27^Al, ^51^V, ^52^Cr, ^55^Mn, ^56^Fe, ^59^Co, ^60^Ni, ^63^Cu, ^66^Zn, ^75^As, ^78^Se, ^88^Sr, ^95^Mo, ^111^Cd, ^137^Ba, ^208^Pb, and ^201^Hg. Since no certified reference materials (CRM) of the brain tissue are available, the analytical method was assessed with the standard addition method.

### Brain and blood neurohormones

The levels of dopamine, corticosterone, cortisol, cortisone, serotonin, were determined in the blood and brain.

The isolation was carried out using C18 extraction columns (500 mg) conditioned by washing sequentially with methanol, water, and 0.01 M ammonium carbonate buffer, pH 9.3. The material was aliquoted (200 μl of plasma, 200 mg of homogenised tissue) with the addition of an internal standard (IS) of 10 μl of deuterated kynurenic acid at a concentration of 1 μg/ml. Next, 1.5 l of 0.01 M ammonium carbonate buffer pH 9.3 was added, thoroughly mixed, and centrifuged. The supernatant was collected and applied onto the extraction column. The matrix was washed with 2 ml of buffer and then the columns were dried. Elution was carried out with the use of 2 ml of methanol with 0.5 M acetic acid (9:1 v/v). Next, the extracts were concentrated by evaporation to dryness and dissolved in 50 µl of the eluent.

Quantification was performed with the method of standard addition to the analysed material. Standards of the analysed compounds were added to the measured samples at concentrations corresponding to the 10 ng/ml, 50 ng/ml, and 100 ng/ml concentrations in the material. Additionally, other samples were prepared without the addition of analytes. Each sample was prepared in duplicate. Next, the dependence of the concentration on the analyte surface area and the internal standard ratio was plotted, and the concentration in the material was determined by extrapolation of the calibration curve to the point of intersect with the abscissa axis.

The selected analytes were determined using high-performance liquid chromatography (HPLC 1260 Agilent Technologies, Germany) coupled with a triple quadrupole mass spectrometer (QqQ 6460, Agilent Technologies, USA). The compounds were separated on a thermostatted (40 °C) chromatography column (Poroshell 120 EC-C18 column 3.0 × 100 mm; 2.7 μm; Agilent Technologies, USA) using eluent A: water with 0.1% formic acid and B: acetonitrile in gradient B: 0 min—5%, 15 min—95%, and 17 min—95% at a flow of 0.5 ml/min. The return to the initial parameters was set at 2 min at a flow of 0.8 ml/min. The mass spectrometer worked in the electrospray ionisation (ESI) mode. The source parameters are presented in Supplementary Table S1. The qualitative and quantitative analyses were performed based on the selected reaction monitoring (MRM/SRM) method. The ESI–MS/MS parameters and retention times for the selected compounds are presented in Supplementary Tables S2 and S3.

The limits of detection and quantification were calculated from the calibration curve equation. The ratio of the sum of the concentration determined for the blank sample and three-fold standard deviation for these samples and the slope of the straight line (LOD) was calculated. The ratio of the sum of the concentration determined for the blank and ten-fold standard deviation for these samples and the slope of the straight line (LOQ) was calculated. Ten blank samples were analysed. The reproducibility was presented as the coefficient of variation determined as the ratio of the standard deviation of the concentration of three calibration samples and the mean value of the concentration. The recovery was determined as the ratio of the concentration determined in the sample enriched with the analytes before the extraction to the concentration determined in the sample enriched after the extraction. The reproducibility and recovery were tested at two calibration levels—10 ng/ml (10 ng/g) and 100 ng/ml (100 ng/g). Three replications were performed for each concentration.

### Haematological analyses

Each blood sample for determination of haematological parameters was immediately poured inside a sample bottle containing the disodium salt of ethylene diaminetetra-acetic acid (EDTA) anticoagulant at the rate of 2 mg/ml of blood^73^. The white blood cell (WBC) count was determined in whole blood samples with the method proposed by Feldman et al.^74^. The percentage composition of white blood cells (leukogram), i.e. the number of heterophils (HET), lymphocytes (LIM), monocytes (MON), eosinophils (EOZ), and basophils (BAZ), was determined by staining blood smears with the Pappenheim method. The numerical ratio of heterophils to lymphocytes was calculated as well.

### Intestinal microbiota

From each Zk and H-33 hen, intestinal contents were collected from three sections: small intestine, caecum, and large intestine into sterile containers and transported in thermobags to the laboratory. Next, 10-g aliquots of the intestinal contents were weighed, inoculated with 9 ml of Ringer’s solution supplemented with Tween 80, and homogenised. Decimal dilutions of each sample were made in accordance with PN-EN ISO 6887-1. Each dilution (100 µl) was plated on sterile solid media and incubated in accordance with relevant standards.

The following parameters were determined in the material:

- total bacterial count—culture on tryptic-soybean (TSA) medium. The material was incubated for 72 (± 3) hours in a thermostat (30 ± 2 °C) in accordance with PN-ISO 4833-2:2013-12^75^,
- total number of yeasts and moulds—culture on agar with dichlorane and 18% addition of glycerol (DG 18). The material was incubated for 5–7 days at 25 ± 1 °C in accordance with PN-ISO 21527-1:2009^76^,
- total number of coliform microorganisms—inoculation on a medium with violet red bile lactose agar (VRBL). The material was incubated for 24 ± 2 h in a thermostat (37 ± 2 °C) in accordance with PN-ISO 4832:2007^77^,
- total number of *Escherichia* *coli*—inoculation on tryptone-bile glucuronide (TBX) medium. The material was incubated for 24 h in a thermostat (44 ± 3 °C) in accordance with PN-ISO-16649-2^78^,
- total number of *Clostridium* *perfringens* microorganisms—inoculation of the samples on a sulfite-tryptose cycloserine (TSC) medium. The material was incubated for 20 ± 2 h at 37 °C (± 1 °C) in anaerobic conditions using the GasPak Plus System (Anaerobic System Envelopes with Palladium Catalyst, BD BBL) in accordance with PN-EN ISO 7937^79^,
- total number of lactic acid bacteria of the genus *Lactobacillus*—culture on MRS agar for 3–5 days at 30 °C (BTL Ltd., Łódź, Poland),
- number of *Listeria* spp.—primary (semi-Fraser broth; Biomaxima) and secondary (Fraser broth; Biomaxima) cultures and inoculation of the material on a selective medium (Chromogenic Listeria) in accordance with PN-EN ISO 11290-1:2017-07^80^,
- the presence of *Salmonella*—culture on SS agar (Salmonella–Shigella and XLD), (BTL Ltd., Łódź, Poland) for 24 h at 37 °C. Final identification was carried out using API tests (BioMerieux, Marcy l’Etoile, France) and polyvalent sera (Biomed Inc., Kraków, Poland)^81^.

The research material was inoculated on each medium in duplicate. The result was presented as the decimal logarithm of colony forming units (CFU) per 1 g of material (Log10 CFU/g).

### Statistical analyses

Data were statistically analysed using the UNIVARIATE, ANOVA, GLM, and NPAR1WAY procedures of SAS software (Statistical Analysis System, 9.4). Normal distribution of the data was examined using the Shapiro–Wilk test and the equality of variance was assessed with the Brown–Forsythe test. The differences in the numbers of neurons between breeds, level of microelements, and blood parameters were tested with ANOVA and the Tukey HSD test as a correction for multiple comparisons. The number of selected intestinal microorganisms was analysed using the two-way analysis of variance with interaction (intestinal segment × breed) and the Tukey HSD test. The differences were considered statistically significant at p < 0.05. Asymmetry of distributions was found in the case of the number of neurotransmitters, and the non-parametric Mann–Whitney *U* test was applied for the ranked data.

## Supplementary Information

Supplementary Table S1.

Supplementary Table S2.

Supplementary Table S3.

## Data Availability

The datasets generated and/or analyzed during the current study are available from the corresponding author on reasonable request.
