# Supplementary material for: Investigation of structural and neurobiochemical differences in brains from high-performance and native hen breeds
Source: Sci Rep. 2023 Jan 5;13:224. doi: 10.1038/s41598-023-27517-3 (PMC9816186; doi:10.1038/s41598-023-27517-3)
Supplement: Supplementary file 1 — Supplementary Table S1. [file 41598_2023_27517_MOESM1_ESM.docx]

**Supplementary Table S1.** Electrospray ionisation (ESI) parameters.

| **Gas temperature** | 300°C |
| --- | --- |
| **Gas flow** | 5 l/min. |
| **Nebuliser** | 45 psi |
| **Sheath gas temperature** | 250^o^C |
| **Sheath gas flow** | 11 l/min |
| **V Cap** | 3500 V positive |
| **Nozzle voltage** | 500 V |
