# Supplementary material for: Investigation of structural and neurobiochemical differences in brains from high-performance and native hen breeds
Source: Sci Rep. 2023 Jan 5;13:224. doi: 10.1038/s41598-023-27517-3 (PMC9816186; doi:10.1038/s41598-023-27517-3)
Supplement: Supplementary file 2 — Supplementary Table S2. [file 41598_2023_27517_MOESM2_ESM.docx]

**Supplementary Table S2.** ESI-MS/MS parameters and retention time of the analysed compounds.

| **Compound** | **Precursor ion** | **Fragmentation ion** | **Fragmenter** | **Collision energy** | **Retention time** **[min]** |
| --- | --- | --- | --- | --- | --- |
| Dopamine | 154.1 | 137  91.1 | 104 | 4  24 | 1.45 |
| Corticosterone | 347.2 | 121  91 | 148 | 24  64 | 9.68 |
| Cortisol | 363.2 | 121  91.2 | 148 | 39  72 | 8.62 |
| Cortisone | 361.2 | 163.1  91.1 | 148 | 24  80 | 8.70 |
| Serotonin | 177 | 160.1  115 | 60 | 4  32 | 2.36 |
| Kynurenic acid –D5 | 195.1 | 149.1  121.1 | 104 | 16  36 | 4.84 |
