# Supplementary material for: Investigation of structural and neurobiochemical differences in brains from high-performance and native hen breeds
Source: Sci Rep. 2023 Jan 5;13:224. doi: 10.1038/s41598-023-27517-3 (PMC9816186; doi:10.1038/s41598-023-27517-3)
Supplement: Supplementary file 3 — Supplementary Table S3. [file 41598_2023_27517_MOESM3_ESM.docx]

**Supplementary Table S3.** ESI-MS/MS Validation parameters.

| **Parameter** | | **Dopamine** | | **Corticosterone** | | **Cortisol** | | **Cortisone** | | **Serotonin** | |
| --- | --- | --- | --- | --- | --- | --- | --- | --- | --- | --- | --- |
|  |  | *Plasma* | *Brain* | *Plasma* | *Brain* | *Plasma* | *Brain* | *Plasma* | *Brain* | *Plasma* | *Brain* |
| **LOD [plasma - ng/ml, brain - ng/g]** | | 1.15 | 1.45 | 0.07 | 0.45 | 0.05 | 0.20 | 0.03 | 0.22 | 0.02 | 0.18 |
| **LOQ [plasma - ng/ml, brain - ng/g]** | | 2.93 | 3.99 | 0.16 | 1.28 | 0.12 | 0.47 | 0.07 | 0.60 | 0.06 | 0.48 |
| **Reproducibility**  **[%]** | 10 ng/ml (g) | 19.79 | 0.76 | 1.13 | 11.83 | 0.31 | 0.05 | 17.35 | 4.52 | 10.72 | 9.28 |
|  | 100 ng/ml (g) | 13.16 | 9.80 | 6.59 | 10.50 | 9.05 | 4.08 | 11.96 | 3.26 | 6.34 | 1.50 |
| **Recovery**  **[%]** | 10 ng/ml (g) | 49.11 | 83.33 | 80.30 | 94.90 | 90.66 | 90.38 | 97.72 | 88.10 | 96.16 | 87.89 |
|  | 100 ng/ml (g) | 77.13 | 85.20 | 97.31 | 77.23 | 92.62 | 88.27 | 92.34 | 86.92 | 84.87 | 93.53 |
